# The involvement of vocal cords in rheumatoid arthritis: a clinical case

**DOI:** 10.11604/pamj.2019.34.102.20490

**Published:** 2019-10-21

**Authors:** Meryem Eddaoudi, Samira Rostom, Bouchra Amine, Rachid Bahiri

**Affiliations:** 1 Service of Rheumatology A, El Ayachi Hospital, University Hospital Center Ibn Sina, Rabat-Salé, Morocco

**Keywords:** Rheumatoid arthritis, vocal cords inflammation, tracheotomy

## Abstract

Rheumatoid arthritis rarely involves the cricoarytenoid joint, symptoms of varying severity ranging from foreign body sensation, fullness or tension in the throat, hoarseness, odynophagia, speech or cough pain to stridor and respiratory distress during bilateral paralysis of the vocal cords. We are reporting a case of rheumatoid arthritis with bilateral involvement of the vocal cords. The diagnosis was clinically made and confirmed by endolaryngoscopy, responding to antirheumatic treatment but coming to the stage of permanent tracheotomy.

## Introduction

Rheumatoid arthritis is an autoimmune disease affecting 3% of the adult population [1,2], characterized by joint and extra-articular manifestations. All joints in the body can be affected including the Crico-arytenoid joint secondary to inflammation of the synovial membrane resulting in joint damage and bone destruction [1,3]. The bilateral vocal cord paralysis (PBCV) may be due to various causes such as inflammatory, infectious, prolonged intubation history, neoplastic, etc. Clinical symptomatology ranges from mild dysphonia to life-threatening airway obstruction [2,4]. The laryngeal manifestations of RA are often sub-clinical, benign and resultant of crico-arytenoid arthritis. An acute airway obstruction is possible causing vocal cord immobility and oedema, which requires urgent tracheotomy [5,6].

## Patient and observation

**Figure 1:**
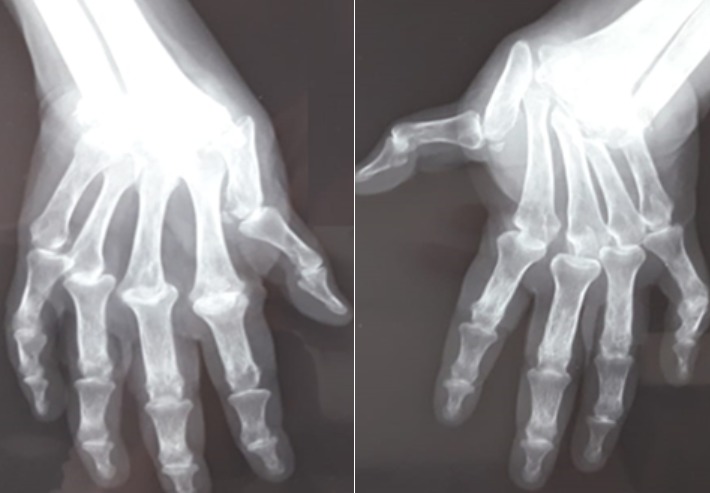
Standard radiograph of the hands of our case: 51-year-old woman with rheumatoid arthritis

We depict a case of a 51 year-old woman undergoing a deforming rheumatoid arthritis and a destructive seropositive that has been evolving for 27 years so far (Figure 1). This patient (with uncontrolled treatment) was taking 12 mg of prednisolone per day. She was not taking the classic DMARDS as a treatment of RA. She displayed a laryngeal dyspnea with progressive installation of dysphonia evolving into a respiratory distress. Intubation was tried, but in vain. Accordingly, an emergency tracheotomy was performed as a rescue procedure. Direct laryngoscopy revealed an ulceration and an inflammation of the posterior commissure of the vocal cord. Biopsies of the posterior commissure reveal non-specific chronic fibro-inflammatory changes and an absence of tumor proliferation. A normal cervical scanner excluding an infectious and tumoral cause. Thus, an inflammatory origin is suspected, particularly the rheumatoid arthritis. The laboratory analyses showed a very important biological inflammatory syndrome CRP (48 mg/dL) and ESR (80 mm/h); it was also a positive serology for anti-CCP (275.3 U.A.) RF (> 512 IU/ml). The rheumatologic examination found 25 painful joints, 8 synovitis and 4 limited joints with rheumatoid arthritis deformities, DAS 28 was at 7.6, while the HAQ score was at 2. The patient received a bolus of solumedrol 500 mg daily for 3 days and the antirheumatic treatment was initiated (methotrexate 15 mg/week). The patient kept under watch for 6 months with permanent tracheotomy, despite the antirheumatic treatment.

## Discussion

In literature, the crico-arytenoid involvement in rheumatoid arthritis has been reported in 17-70% of cases [7], and laryngeal nodule is found in about 20% cases [8]. The involvement of the crico-arytenoid joint is responsible for the symptoms of varying severity ranging from foreign body sensation, fullness or tension in the throat, hoarseness, odynophagia, speech or cough pain to stridor and respiratory distress during bilateral paralysis of the vocal cords [2,9]. The bilateral involvement of the crico-arytenoid joint may be due to an infection of the upper airways, acute inflammation or chronic inflammatory disease such as rheumatoid arthritis [2]. It has been reported in the literature that the vocal cord involvement in patients with RA does not necessarily correspond to the specific laryngeal involvement caused by the disease, represented by cricoarythenoid involvement and rheumatoid nodules of the vocal cords [10]. This is a case of our patient displaying a laryngeal dyspnea with progressive dysphonia evolving to a respiratory distress leading to inflammation and ulceration of the vocal cords without crico-artythenoidal lesions or nodules of the cords. This finding was revealed by direct laryngoscopy and confirmed by biopsies of the posterior commissure showing non-specific chronic fibro-inflammatory changes and an absence of tumor proliferation. Also, the cervical scanner was normal; the fact which excludes infectious and tumoral cause. The department of oto-rhino-laryngoscopy has implicated an inflammatory origin of the involvement of the vocal cords including its rheumatoid arthritis after eliminating the other tumoral, infectious cause. The patient received a bolus of solumedrol 500 mg / day for 3 days and started a treatment for the rheumatoid arthritis (methotrexate 15 mg/week). The response to antirheumatic treatments in patients with crico-arythenoid arthritis is variable although there is a significant improvement in the extra-laryngeal manifestations of rheumatoid arthritis. A surgical treatment can also be proposed in these patients with arytenoidectomy and vocalization [2]. Here, we have described a case of severe destructive rheumatoid arthritis with an inflammatory and ulcerated appearance of the 2 vocal cords at the post-nasofibroscopy commissure confirmed by biopsies that demonstrated non-specific chronic fibro-inflammatory changes and an absence of a growing tumor. The patient responded to an RA treatment though it was too late onto the stage of permanent tracheostomy.

## Conclusion

The Laryngeal symptoms which are common in RA patients can lead to symptoms of varying severity ranging from respiratory distress to emergency tracheotomy. The alteration of the vocal cords outside the crico-arythenoid arthritis and rheumatoid nodules witnessed in RA has been reported in the literature; our patient is a case in point. Antirheumatic drugs are first-line treatment laryngeal alterations with variable results.
